# Rotating and stacking genes can improve crop resistance durability while potentially selecting highly virulent pathogen strains

**DOI:** 10.1038/s41598-020-76788-7

**Published:** 2020-11-12

**Authors:** Rémi Crété, Rodrigo Neto Pires, Martin J. Barbetti, Michael Renton

**Affiliations:** 1 School of Agriculture and Environment, Faculty of Science, The University of Western Australia, Crawley, WA 6009 Australia; 2 School of Biological Sciences, Faculty of Science, The University of Western Australia, Crawley, WA 6009 Australia; 3 The UWA Institute of Agriculture, The University of Western Australia, Crawley, WA 6009 Australia

**Keywords:** Agricultural genetics, Virulence, Plant sciences, Ecological modelling

## Abstract

Rotating crop cultivars with different resistance genes could slow the evolution of virulent strains of fungal pathogens, but could also produce highly virulent pathogen strains. We present a new model that links polycyclic pathogen epidemiology and population genetics in order to predict how different strategies of rotating cultivars with different resistances will affect the evolution of pathogen virulence and the breakdown of crop resistance. We modelled a situation where there were four different resistance genes that can be deployed within each crop cultivar, and four virulence genes that may be present within the pathogen. We simulated four different rotational management strategies: (i) no rotation; (ii) a different gene every year; (iii) a different gene every 5 years; and (iv) a different combination of two stacked genes each year. Results indicate that rotating cultivars can lead to longer periods of disease suppression but also to the selection of highly virulent strains. The efficacy and relative advantage of different resistant cultivar rotation strategies depended on the fitness penalties, initial virulence allele frequencies, and ability of non-virulent pathogen genotypes to grow and reproduce on resistant cultivars. By capturing the essential processes involved, our model provides a useful new tool for investigating the evolutionary dynamics of pathogen virulence and crop resistance breakdown.

## Introduction

In agrosystems, farm stakeholders deploy an array of crop management practices to control the spread of pathogen populations and the severity of disease incidence. Reliable crop protection management practices should ideally, achieve efficient and durable epidemic control. The efficiency of such strategies depends on pathogen biology and population size (i.e. epidemiology dynamics) while their durability (i.e. the persistence of their efficacy in time and space) depends on adaptation dynamics in pathogen populations (i.e. evolutionary dynamics)^1–4^. Modern and more intensive agricultural production systems increasingly employ new technologies aiming to facilitate crop management and ultimately enhance crop productivity^5–7^. Fast paced modernisation of agricultural systems combined with significant resource input greatly impact evolutionary trajectories of pathogens and pests^8^. These practices may lead to unintended outcomes, such as disease epidemics that are more severe and harder to control^3^. Moreover, the reduction in plant genetic and physiological diversity, and the increased homogeneity of modern cropping systems has disrupted selective pressures and the natural co-evolution of plant-pathogen systems^6,9,10^. Unwanted consequences arise, and the rate at which crop pathogens evolve and overcome host resistance is key^9,11^, as the development and deployment of resistant crop varieties is time and resource consuming^12^.

Typically, management strategies rely on disease control methods such as pesticide applications, deep tillage of crop residues, the use of resistant cultivars, specific sowing period, crop rotations, cultivar mixtures^9,13–17^. Breeding and deploying resistant crop varieties is one of the most used strategies to control crop pathogens^12,18,19^. However, crop resistance can be circumvented by adaptation of the pathogen if the same type of resistance is deployed over several years, as occurs with *Leptosphaeria maculans* populations (stem phoma canker—Blackleg disease)^20,21^. Shifts in pathogen species and virulence diversity^22,23^ can also lead to resistance breakdown. This also includes the development of new pathogen pathotypes, such as occurs with other Brassicaceae pathogens like *Sclerotinia sclerotiorum*^24^, *Hyaloperonospora brassicae*^25^, *Albugo candida*^26^, *Neopseudocercosporella capsellae*^27^ and, *Alternaria* spp.^28^. The same occurs for many other crop-pathogen combinations, such as pulse crop pathogens like *Phytophthora sojae*^29^ and legume forage pathogens like *Phytophthora clandestina*. Thus, crop management strategies targeting resistance durability and epidemiological control have to be developed and deployed to avoid and/or delay the evolution of virulence and the breakdown of crop resistance^2,6,20,30,31^.

There is a need to consider under what conditions different management strategies will lead to the the accumulation of virulent genes in certain pathogen genotypes (i.e. ‘super-virulent strains’), given the limited available area (surface) for infection and, consequently, competition between pathogen strains. The accumulation of virulence genes into highly virulent races could be considered as an adaptive response of pathogen populations to the selection exerted by successive resistant hosts and cropping practices^32^. Furthermore, some localities are more prone to resistance breakdown events than others; for example, the mean number of virulence alleles per *L. maculans* isolate is higher in Australia (5.11 virulence alleles) than in Europe (4.33) and Canada (3.46)^21^. Super-virulent pathogen strains could then overcome resistance genes and/or management strategies, highlighting the risk of resistance breakdown events particularly for cropping regions with higher number of virulence genes^21^. A convenient example is the simultaneous failure of two major host resistance genes in hybrid oilseed rape cultivars in 2012, in South Australia^33^. Therefore, while rotation and/or stacking of resistance genes are efficient crop management strategies, their deployment can potentially lead to the development of ‘super-virulent’ strains.

Mechanistic models^34^, whether stochastic or deterministic, are invaluable tools for integrating key disease processes, such as epidemiological and evolutionary dynamics. The use of modelling approaches allows for the assessment of crop management practices before implementation and deployment in scale. Strategies to maximise durability of resistance genes in crop cultivars should both limit the selection of more virulent pathogen strains while also reducing population sizes^18,35^. A number of mechanistic models have been developed and tested for fungal pathogens and other pests (e.g. Blackleg^36^, citrus tristeza virus^37^, anthracnose^38^, yellow rust of wheat^39^, forest gap generated by bark beetles species^40^ and apple scab^41^). Historically, models have focused on evolution of resistance to chemical pesticide applications, rather than evolution of virulence^42^. However, recent modelling approaches have successfully incorporated evolutionary dynamics of plant pathogens^10,17,31,43–45^ even whilst assuming non-sexual reproduction. Sexual recombination is likely to be important when investigating evolutionary dynamics^6,46^. We are unaware of modelling approaches that take into account secondary fungal pathogen spread and infection (i.e. a policyclic disease) and successfully incorporate sexual recombination into models, the exception being the SIPPOM-WOSR for Blackleg disease management^35,47,48^. The SIPPOM-WOSR was built by integrating multiple sub-models and represents many bio-physical variables at a relatively high degree of detail and realism^47,48^. However, model complexity comes at the price of many parameters and increasing interacting processes culminating in high sensitivity to parameter variations^34^. It seems likely that while factors such as the specific weather conditions and soil characteristics will be important drivers defining disease epidemic development over a particular season, the inter- and intra-seasonal variation is likely to be less important to long-term dynamics which is the focus of this work.

**Figure 1:**
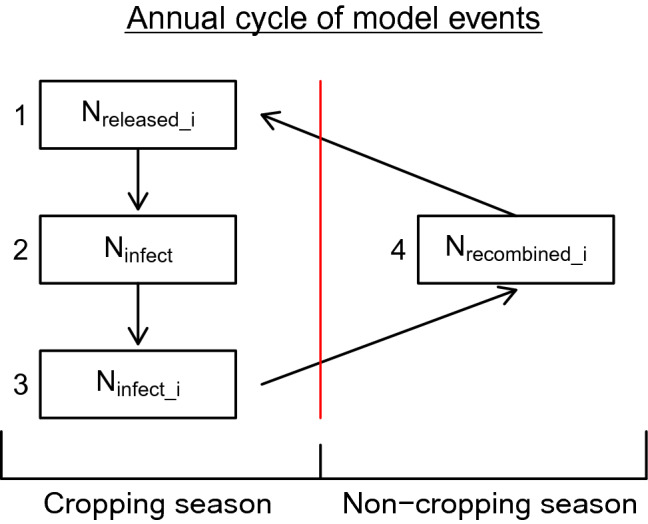
The modelled annual cycle of how the density of different pathogen strains changes over a number of years, as they interact with different resistant cultivars planted in a field. First, the total number of spores released is simulated independently for each strain *i*. Second, the total number of infections is calculated, with limitations due to space or the number of possible infection sites. Third, this total number of infections is apportioned between the different strains, while accounting for any fitness penalties and/or cultivar resistance. Fourth, sexual recombination (mating) is simulated. The symbol $N_i$ represents the number of a given strain *i*, whereas *N* with the *i* missing means the total for all strains.

**Figure 2:**
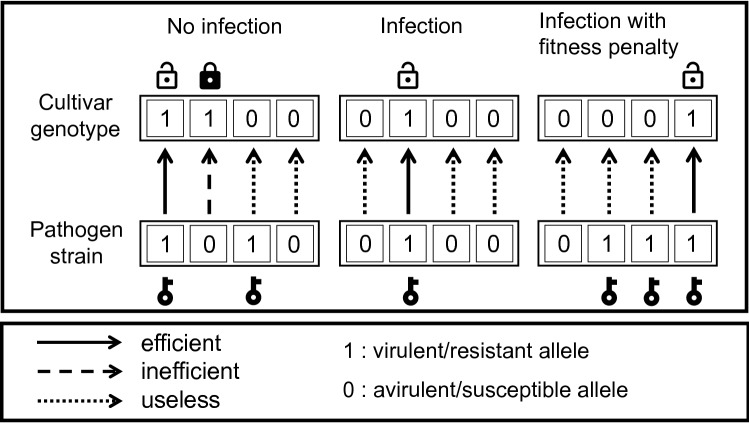
Example illustrations of the different kinds of cultivar-strain interaction for $\nu = 4$: on the left, no infection because the pathogen strain genotype does not overcome the cultivar genotype (the strain does not have a ‘key’ for each resistance ‘lock’ on the cultivar, and so the resistance lock stays locked); in the middle, infection and high pathogen growth rate, because the pathogen has the minimum number of genes needed to overcome the cultivar’s resistance genes (only the necessary keys for the resistance locks); on the right, infection and lower pathogen growth rate, because the pathogen has more than the minimum number of genes needed to overcome the cultivar’s resistance genes (more than just the necessary keys for the resistance locks).

Therefore, we concluded that no existing model was suitable for addressing our aim of investigating—at the long-term and general level—how strategies of rotating resistant cultivars would influence evolution of pathogen virulence and the durability of crop resistance. We develop a spatially implicit model that accounts for important processes involved in evolution of pathogen virulence over a number of seasons, focusing in details important to long-term dynamics. In particular, we wanted the model to represent changes in the genetic structure of pathogen populations over time as a result of different strategies for rotating resistant crop cultivars and resistance genes, while accounting for sexual recombination and competition between pathogen strains for limited infection sites (for details see Figs. 1, 2). Moreover, we wanted the model to not only be capable of being applied to Blackleg disease, but also more generally to other diseases. We then used the model to address the following specific questions:

(i) How do initial pathogen densities and virulence allele frequencies influence the selection of ‘super-virulent’ strains under particular resistant cultivar rotation strategies?
(ii) Given a fixed number of resistance genes, what is the best resistant cultivar rotation strategy to delay evolution of virulence and breakdown of cultivar resistance?
(iii) Is it possible, under certain conditions, to delay the breakdown of resistance indefinitely, even if the same crop is planted every year?

## Methods

### Overview of the model

This model simulates the population and evolutionary dynamics of different pathogen strains, as they interact with different crop resistant cultivars planted in a single field over successive years. We assume one cultivar is planted each year and we consider a field divided into a finite number *m* of spatial units (representing limited spaces for infections, or potential lesion sites), in which the spatial aspect is implied rather than explicitly represented. For each year during the cropping season, a number of pathogen spores are released from the infested crop residues, it then lands on the crop plants leading to infections (Fig. 1). These infections are apportioned between the different pathogen strains depending on their previous abundance and interactions with the crop cultivar. At the end of the year, during the non-cropping season, these strains are assumed to sexually recombine in the crop residue. The number of spores released and the number of infections are considered as random variables. We denote these both quantities with an uppercase letter (for example *N*) in general sense, while their particular realisation or draw in the simulation will be noted with a lowercase letter (for example *n*). The model was developed using the R Language and Environment for Statistical Computing^49^.

**Figure 3:**
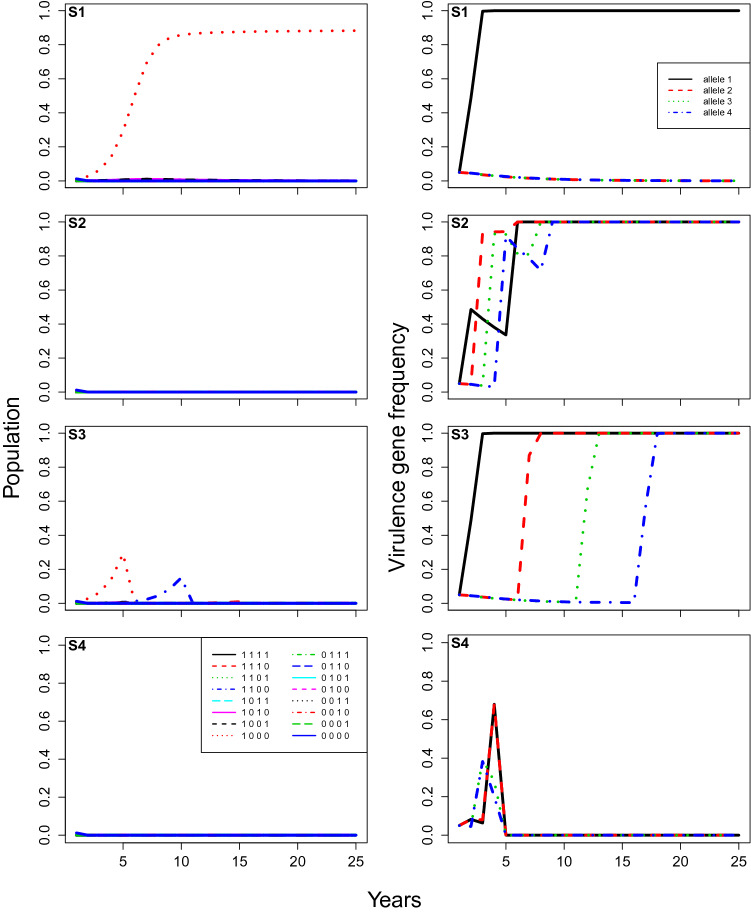
**Case 1**, model predictions of total infection by each pathogen genotype (proportion of total locations infected, left), and the corresponding frequencies of each virulent allele (right) changing over time under different rotation strategies (From top to bottom: (**S1**) no rotation; (**S2**) rotation every year; (**S3**) rotation every 5 years; and (**S4**) rotation every year with stacked resistance genes). The parameters are at baseline values: the initial frequency of each virulent allele equals $5\%$, the fitness modifier is set at 0.9, the modifier of increase rate for non-virulent strains equals 0.05, and the initial amount of inoculum represents $10 \%$ of available locations.

**Figure 4:**
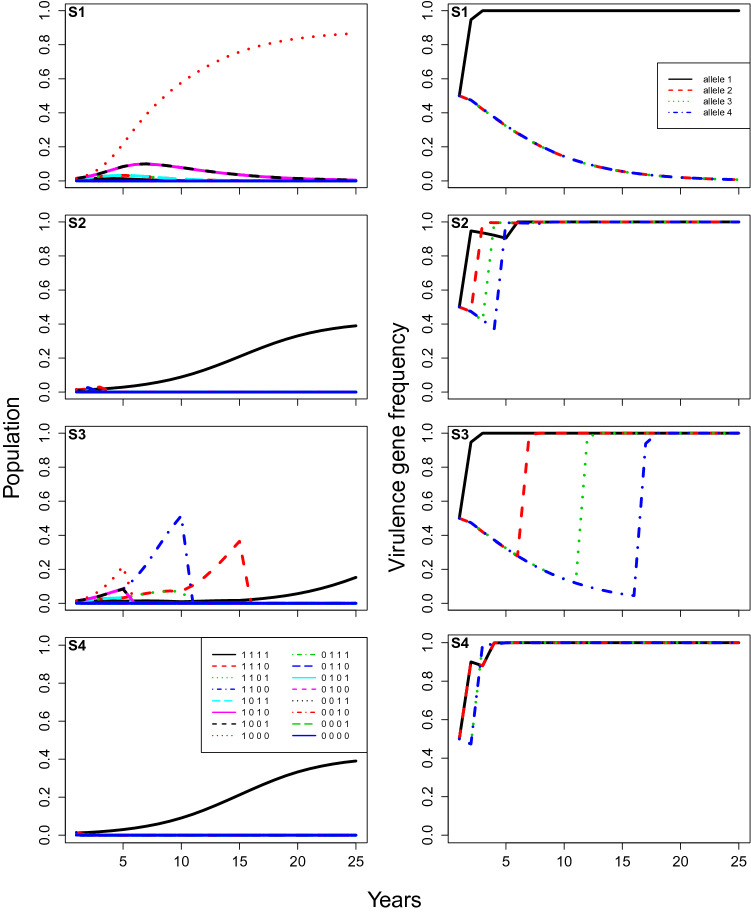
**Case 2**, model predictions of total infection by each pathogen genotype (proportion of total locations infected, left), and the corresponding frequencies of each virulent allele (right) changing over time under different rotation strategies (from top to bottom: (**S1**) no rotation; (**S2**) rotation every year; (**S3**) rotation every 5 years; and (**S4**) rotation every year with stacked resistance genes). The initial frequency of each virulent allele equals $50\%$, and other parameters are at baseline values: the fitness modifier is set at 0.9, the modifier of increase rate for non-virulent strains equals 0.05, and the initial amount of inoculum represents $10 \%$ of available locations.

We applied the general model described above to a specific situation with four genes of interaction where there are four different resistance genes that may or may not be deployed within each crop cultivar, and four virulence genes that may or may not be present within each pathogen strain. We assume that the presence of each virulence gene reduces the fitness of a strain independently. Specifically, for each strain *i*, we define the fitness of that strain $\delta _i = \delta ^{n_{vir,i}}$, where $n_{vir,i}$ is the number of virulence alleles present in strain *i*, and $\delta$ is a fixed model parameter with potential values between zero and one (Table 1).

**Table 1:** Baseline model parameter values used for our analysis, with alternative values shown in parentheses.

Parameters	Description	Values
*m*	Carrying capacity (maximum number of lesions)	10,000,000
*Init*.*path*	Initial quantity of pathogen	1,000,000
*Init*.*freq*	Initial frequency of virulent allele	0.05 (or 0.5)
*nb*.*years*	Number of years	25
*nu*	Number of loci	4
*I*	Number of strains	16
$\alpha$	Disease increase rate	3
$\delta$	Fitness modifier associated with virulence alleles	0.9 (or 0.7)
$\epsilon$	Modifier of increase rate for non-virulent strains	0.05 (or 0.5)

We first set the model parameter values to define a baseline situation where there is a relatively small fitness penalty for virulence alleles (i.e. $\delta$ is very close to 1, where the value 1 means no penalty); the pathogen has a relatively low ability to reproduce if it does not carry effective virulence genes (i.e. low value for $\epsilon$, in this baseline situation equal to 0.05); the initial virulence allele frequency (*Init*.*freq*) is relatively low, reflecting a low historical selection pressure and lastly the initial quantity of pathogen (*Init*.*path*) is also low at $10 \%$ of carrying capacity (Table 1). We then considered and compared four different strategies for rotating resistant crop cultivars:

1. No rotation, the same cultivar with only one gene of resistance is employed every year;
2. A cultivar with a single gene of resistance is employed each year, and the gene of resistance in the cultivar is changed every year, giving a 4-year rotation;
3. A cultivar with a single gene of resistance is employed each year, and the gene of resistance in the cultivar is changed every 5 years, giving a 20-year rotation; and
4. A cultivar with two genes of resistance (i.e. pyramided resistance) is employed each year, and the genes of resistance in the cultivar are changed every year, giving a 2-year rotation

We then investigated how different parameterisations of the model would interact with the selected rotation strategies. We develop four cases in addition to the baseline case described above:

1. Baseline scenario (Table 1).
2. Baseline scenario, except for *Init*.*freq* which was increased from 0.05 to 0.5.
3. Baseline scenario, except for *Init*.*freq* which was increased from 0.05 to 0.5 and $\delta$ which was decreased from 0.9 to 0.7.
4. Baseline scenario, except for *Init*.*freq* which was increased from 0.05 to 0.5, $\delta$ which was decreased from 0.9 to 0.7 and $\epsilon$ which was increased from 0.05 to 0.5.
5. Baseline scenario, except for $\epsilon$ which was increased from 0.05 to 0.5.

### Genetics

Cultivar and pathogen strain are both defined through their genotype being restricted to a specific set of interaction genes (loci) related to resistance (for the cultivar) or virulence (for the pathogen). Each gene has two versions (alleles): virulence or avirulence allele for the pathogen and resistance or susceptibility for the cultivar. Virulence and resistance are represented with a 1 and avirulence and susceptibility are represented with a 0 (Fig. 2). If we call ${\mathcal {I}}$ the set of strains and if $\nu$ genes of interaction are involved, then the total number of strains will be $\left| {\mathcal {I}}\right| = 2^{\nu }$. During the infection process, after pathogen spores land on the cultivar, an interaction factor $\beta (i,c)$ defines the relative rate at which strain *i* can reproduce within a field of cultivar *c*, for each strain and cultivar combination (Fig. 2). We consider that a strain overcomes the cultivar genotype when the strain has a virulence allele for every resistance allele of the cultivar (Fig. 2), in which case $\beta (i,c)=1$, indicating maximum reproduction rate. Otherwise, if the strain does not have a virulence allele for every resistance allele of the cultivar, $\beta (i,c)= \epsilon$, where $\epsilon$ is a model parameter with constant value $0 \le \epsilon < 1$, indicating a less-than-maximum reproduction rate. As such, $\epsilon$ is the model parameter modifying the growth and reproduction of pathogen strains not carrying multiple virulence alleles (e.g. 0100) and/or an avirulent pathogen strain (e.g. 0000) (Fig. 2). Accordingly, lower (closer to 0) $\epsilon$ values represent reduced ability to grow and reproduce in pathogen strains with increasing number of avirulence alleles. Moreover, any strain *i* with one or more virulence genes is also assumed to suffer a fitness penalty $\delta _i$ depending on the number of genes involved. Together these interaction factors make a cultivar-strain interaction matrix $B = (\beta (i,c))$. This code and method for modelling resistance and virulence interactions (without fitness penalty) is similar to those in previous studies^48,50^.

**Figure 5:**
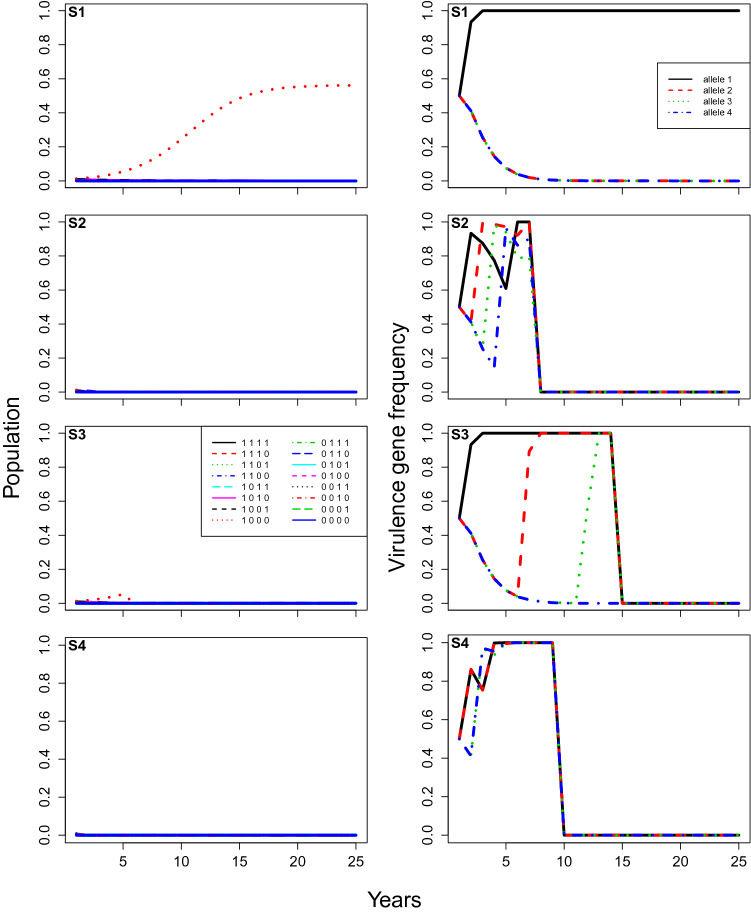
**Case 3**, model predictions of total infection by each pathogen genotype (proportion of total locations infected, left), and the corresponding frequencies of each virulent allele (right) changing over time under different rotation strategies (from top to bottom: (**S1**) no rotation; (**S2**) rotation every year; (**S3**) rotation every 5 years; and (**S4**) rotation every year with stacked resistance genes). The fitness modifier is set at 0.7, the initial frequency of each virulent allele equals $50\%$, and other parameters are at baseline values: the modifier of increase rate for non-virulent strains equals 0.05, and the initial amount of inoculum represents $10 \%$ of available locations.

**Figure 6:**
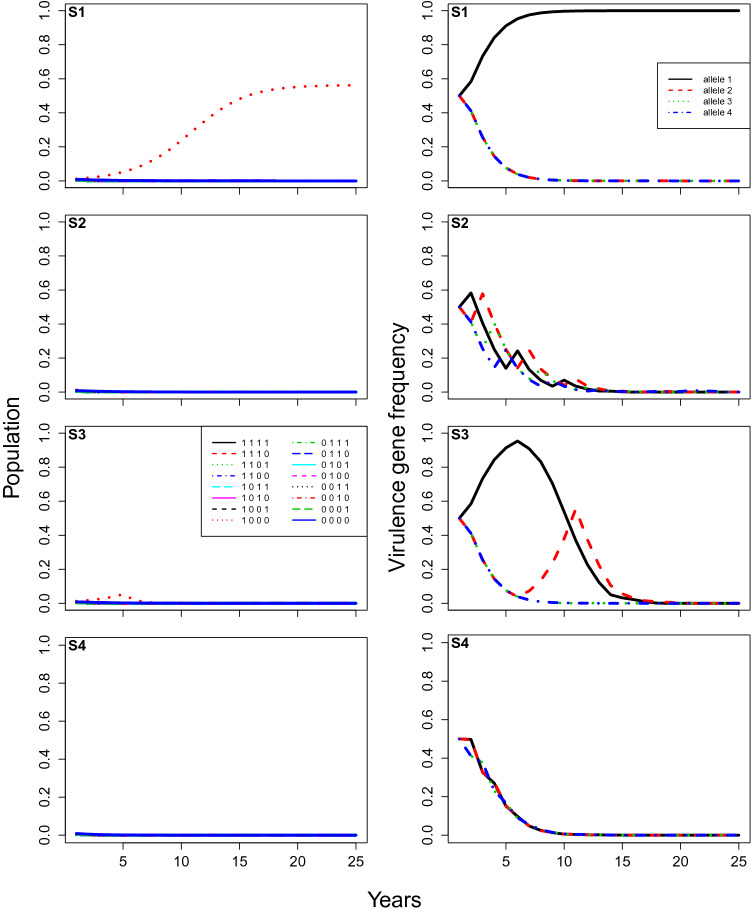
**Case 4**, model predictions of total infection by each pathogen genotype (proportion of total locations infected, left), and the corresponding frequencies of each virulent allele (right) changing over time under different rotation strategies (from top to bottom: (**S1**) no rotation; (**S2**) rotation every year; (**S3**) rotation every 5 years; and (**S4**) rotation every year with stacked resistance genes). The fitness modifier is set at 0.7, the initial frequency of each virulent allele equals $50\%$, the modifier of increase rate for non-virulent strains equals 0.5, and other parameters are at baseline values: the initial amount of inoculum represents $10 \%$ of available locations.

#### Initial genetic structure of pathogen population

At the start of each case, we define the initial proportion of each pathogen genotype using the equation:

1 $$\begin{aligned} strains.init = Init.freq^{nr}\left( 1-Init.freq\right) ^{4-nr} \end{aligned}$$

where *strains*.*init* is the initial proportion of each pathogen genotype; *Init*.*freq* is the frequency of the virulent genes as set by each case and *nr* is the number of virulent genes present in a given pathogen genotype. We then used a random Poisson distribution generator (rpois function from the stats package in R) to obtain the initial number of spores for each pathogen genotype, where the mean of the Poisson distribution is the proportion of a given pathogen genotype multiplied by the pre-determined pathogen load (*Init*.*path*, Table 1).

### Model dynamics

The annual dynamics (Fig. 1) can be divided into two main phases: the phase of parasitic activity, representing events occurring through the cropping season, and the phase of dormancy, representing events occurring between the cropping seasons. During the phase of parasitic activity, the pathogen produces spores which are spread both through the air (sexual ascospores) and via water splash (asexual conidia). These spores may then infect leaves and stems of the cultivar, resulting in new lesions of different strains. During the phase of dormancy, the pathogen remains within the infected crop residue and sexual recombination occurs. These processes are modelled with four steps, three for the parasitic phase and one for the dormancy phase.

**Figure 7:**
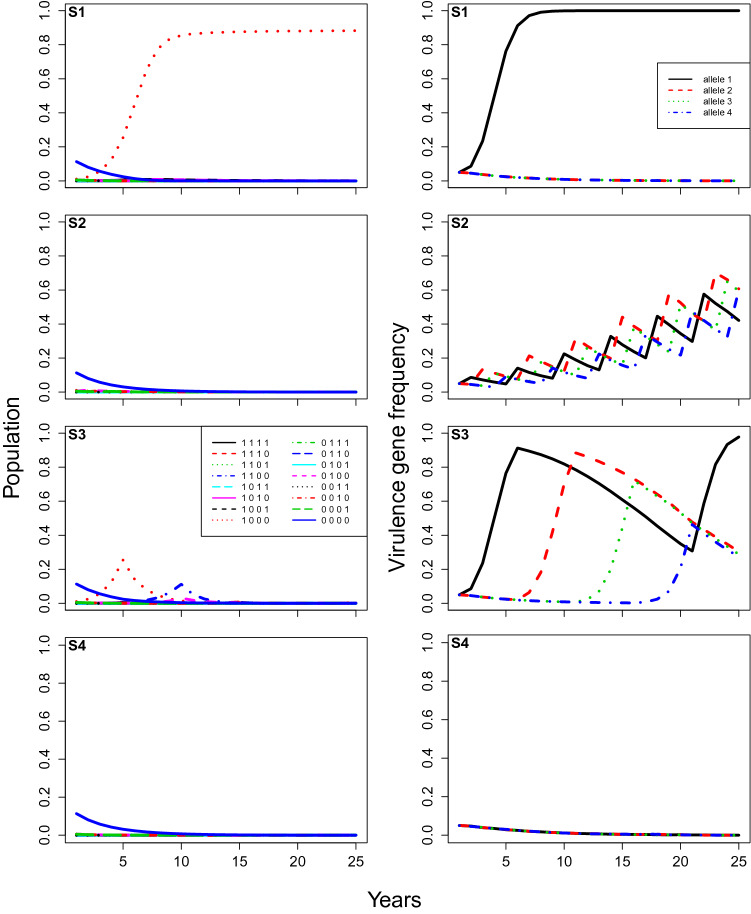
**Case 5**, model predictions of total infection by each pathogen genotype (proportion of total locations infected, left), and the corresponding frequencies of each virulent allele (right) changing over time under different rotation strategies (from top to bottom: (**S1**) no rotation; (**S2**) rotation every year; (**S3**) rotation every 5 years; and (**S4**) rotation every year with stacked resistance genes). The modifier of increase rate for non-virulent strains equals 0.5 and other parameters are at baseline values: the initial frequency of each virulent allele equals $5\%$, the fitness modifier is set at 0.9, and the initial amount of inoculum represents $10 \%$ of available locations.

#### Total amount of spores released

First, the model generates the amount of pathogen spores of each strain that is released, using the equation:

2 $$\begin{aligned} \lambda _{{ released},i}(t) = \alpha . n_{{ recombined},i}(t-1) \end{aligned}$$

where $\lambda _{{ released},i}(t)$ represents the expected dispersed propagule (spore) pressure in the field due to strain $i \in {\mathcal {I}}$ during the year *t*, the parameter $\alpha$ represents the normal rate of growth for the pathogen from 1 year to the next, and $n_{{ recombined},i}(t-1)$ represents the number of spatial units or locations infected by the strain *i* at the end of the previous year and after genetic recombination. The actual quantity of pathogen strain *i* released in the current year, $n_{{ released},i}(t)$ is then simulated as a Poisson random variable:

3 $$\begin{aligned} N_{{ released},i}(t) \hookrightarrow {\mathcal {P}}(\alpha . n_{{ recombined},i}(t-1)) \end{aligned}$$

The infective pressure $\lambda _{{ infected},i}(t)$ is then calculated as:

4 $$\begin{aligned} \lambda _{{ infected},i}(t) = \beta (i,c(t)) . \delta _i . n_{{ released},i}(t) \end{aligned}$$

where $\beta (i,c(t))$ is the interaction factor between the strain *i* and the cultivar *c*(*t*) i.e. the cultivar grown in year *t*, and $\delta _i$ is the fitness penalty for the particular strain *i*.

#### Total number of infections

Second, the model calculates the total number of infected sites, following a binomial distribution:

5 $$\begin{aligned} N_{ infected}(t) \hookrightarrow {\mathcal {B}}\left( m, 1 - \prod _{i = 1}^{2^{\nu }} (1- \rho _i(t))^{n_{{ released},i}(t)}\right) \end{aligned}$$

where $\rho _i(t)$ is the probability that a particular given location (among the *m* possible locations in the field) during year *t*, will have a given spore from strain *i* fall down on it and cause a lesion, and thus $\displaystyle 1 - \prod \nolimits _{i = 1}^{2^{\nu }} (1- \rho _i(t))^{n_{{ released},i}(t)}$ represents the probability that at least one of the $\displaystyle n_{ released}(t) = \sum \nolimits _{i = 1}^{2^{\nu }} n_{{ released},i}(t)$ spores produces a lesion. This equation can be justified in more detail as follows:

$$\begin{aligned}&P({At\; least\; one\; of\; the\; n_{ released}(t)\; spores\; produces\; a\; lesion}) \\&\quad = 1 - P({ No\; released\; spores\; produces\; a\; lesion}) \\&\quad = 1 - \prod _{i = 1}^{2^{\nu }} P({ A\; single\; released\; spore\; of \; strain \; i \; doesn't\; produce\; a\; lesion})^{n_{{ released},i}(t)} \\&\quad = 1 - \prod _{i = 1}^{2^{\nu }} (1 - P({A\; single\; released\; spore\; of \; strain \; i \; produces\; a\; lesion}))^{n_{{ released},i}(t)} \end{aligned}$$

We assume that a spore will fall on any of the *m* specific locations with the same probability independently of its infection capabilities. The number of locations *m* is assumed to be the same for all years whatever the cultivar grown and thus, this probability is independent of the time dimension. Next, we assume that the probability that a spore will induce an infection depends on the interaction factor between the crop cultivar genotype and the pathogen strain $\beta (i,c(t))$ together with the fitness penalty for that strain $\delta _i$ . These assumptions mean that:

$$\begin{aligned} \rho _i(t) = P({a\; spore\; fall\; down\; on\; a\; given\; location\; during \; year\; t\; and\; causes\; a\; lesion}) \\ = P({a\; spore\; falls\; on\; a\; given\; place\; where\; c(t)\; is\; grown })\times \\ P({ the\; spore\; causes\; a\; lesion} \mid { the\; spore\; falls\; on\; a\; place\; where\; c(t)\; is\; grown}) \\ = \frac{1}{m} . \beta (i,c(t)) . \delta _i \end{aligned}$$

#### Number of infections for each strain

Third, the number of infections of each strain is derived from the total number of infections depending on genetic interactions between each strain and crop cultivar being employed that year. Specifically, the total number of infections $N_{ infected}(t) = n_{ infected}(t)$ is distributed among the different strains using the multinomial distribution:

6 $$\begin{aligned} \left( N_{{ infected},1}(t), \ldots , N_{{ infected},2^{\nu }}(t)\right) \hookrightarrow {\mathcal {M}}\left( \frac{\lambda _{{ infected},1}(t)}{\lambda _{ infected}(t)},\ldots , \frac{\lambda _{{ infected},2^{\nu }}(t)}{\lambda _{{ infected}}(t)}, n_{ infected}(t)\right) \end{aligned}$$

where $\displaystyle \lambda _{{ infected}} (t) = \sum \nolimits _{i = 1}^{2^{\nu }} \lambda _{{ infected},i}(t)$. The number of infected sites due to strain *i*, without no loss of generalities, follows then the binomial distribution $\displaystyle {\mathcal {B}}\left( n_{ infected}(t),\frac{\lambda _{{ infected},i}(t)}{\lambda _{{ infected}}(t)}\right)$.

#### Genetic recombination

The fourth step involves simulating the process of sexual recombination, where new quantities of each strain are generated based on the previous quantities of each strain. At the end of the year *t*, we calculate the frequencies $f_j(t)$ of each virulent version of each gene from the different genotypes of strains in the crop stubble. We let the genotype of any new spore be represented by a random vector $\displaystyle G_i(t) = \left( G_{i,1}(t), \ldots ,G_{i,\nu }(t)\right)$, where each $G_{i,j}(t)$ is a Bernoulli random variable $\displaystyle {\mathcal {B}}(1,f_j(t))$. This vector representation of genotype follows the coding illustrated in (Fig. 2). Assuming that strains recombine independently gene by gene, the probability that $G_i(t)$ will be a particular genotype $\displaystyle g_i(t) = \left( g_{i,1}(t), \ldots ,g_{i,\nu }(t)\right)$ is given by:

$$\begin{aligned} p_i(t) = P\left( G_i(t) = g_i(t)\right) &= \prod _{j=1}^{\nu } P(G_{i,j}(t) = g_{i,j}(t)) \nonumber \\ &= \prod _{j=1}^{\nu } f_j(t)^{g_{i,j}(t)} \left( 1 - f_j(t)\right) ^{\left( 1 - g_{i,j}(t)\right) } \end{aligned}$$

We can also confirm that across all possible genotypes these probabilities sum to one:

7 $$\begin{aligned} \sum _{i=1}^{2^{\nu }} P\left( G_i(t) = g_i(t)\right) = \sum _{i=1}^{2^{\nu }} \prod _{j=1}^{\nu } f_j(t)^{g_{i,j}(t)} \left( 1 - f_j(t)\right) ^{\left( 1 - g_{i,j}(t)\right) } = 1 \end{aligned}$$

If we shorten the notation for $P(G_i(t) = g_i(t))$ to be $p_i(t)$ then we can define the recombined version of infected numbers of units of each strain with the following multinomial distribution:

8 $$\begin{aligned} \left( N_{{ recombined},1}(t), \ldots , N_{{ recombined},2^{\nu }}(t)\right) \hookrightarrow {\mathcal {M}}\left( p_1(t),\ldots , p_{2^{\nu }}(t), n_{ infected}(t)\right) \end{aligned}$$

### Poisson, binomial and multinomial distribution

In plant pathology, it is often relevant to model infections by a random variable. Let’s imagine a released spore flying in the air, we can say that this spore will land on a specific leaf and infect it with a given probability *p*, then it won’t with probability $1 - p$ because these are the only two possible events. We can define *Y* a random variable to model the situation. If we say the event $\{Y = 1\}$ represents the success of the event (landing and infection) and $\{Y = 0\}$ represents the failure, with this definition we say that *Y* follows a Bernoulli distribution. The values attributed to the variable depending on the events allow the following generalisation: If we consider *n* spores, each of them realizing an infection on a specific plant area they fell on with the same probability *p*, then we can associate to each spore a Bernoulli distribution $Y_i$ where $i \in \{1,\ldots ,n \}$. If we are interested in the total number of infections occurring on this leaf, assuming the fact that they will happen independently of each other, we can model this situation by the variable $\displaystyle S = \sum \nolimits _{i = 1}^n{Y_i}$, called binomial variable. We can also denote briefly $S \hookrightarrow {\mathcal {B}}(n,p)$, where *n* represents the number of events and *p* the probability of success of each event. Moreover, the Bernoulli variable *Y* is related to binomial distribution in the way that we can write $Y \hookrightarrow {\mathcal {B}}(1,p)$^51,52^.

Usually it is more likely to model such events by a Poisson law rather than binomial law^53,54^. When the number of events is so big that we can approximate it by infinity, and when the probability of success of each event is very small, close to zero, it is possible to link both Poisson and binomial distribution through their respective expectations. So if $$\lim \nolimits _{\begin{array}{c} n \nearrow +\infty \\ p \searrow 0 \end{array}} \ n*p = \lambda ,$$ then if we define $X \hookrightarrow {\mathcal {P}}(\lambda )$, we have $S \xrightarrow {\text {distribution}} X$. Returning to our example, that means that if we have a ‘close to infinity’ number of spores that could fall onto a given plant and infect it with a very small probability *p* for each of them and still acting independently, we can model the total number of infections by both *S* or *X*. Even if there are millions and millions of spores released, this amount is still small compared to infinity, so using *X* is still a modelling approximation. The use of binomial or Poisson laws depends on the complexity of the situation. For example, if the modeller wants to simulate a model where he anticipates 15 infections, they can use $X \hookrightarrow {\mathcal {P}}(15)$ or $S \hookrightarrow {\mathcal {B}}(10000,0.0015)$ or $S \hookrightarrow {\mathcal {B}}(1000000,0.000015)$.

We consider now a situation where the plant is attacked by a big number of spores, but with different genotypes modifying their ability to infect, some strains being more efficient than others. To model this situation, we can use a vector of variables, each component representing the number of successes due to a specific genotype. We can choose a vector of binomial number or Poisson number. If we consider the case of a threshold in terms of available space to be infected (a maximum number of infections for the plant), such that spores of different strains are competing for those places, we suggest using a vector of random numbers that follows a multinomial law. This distribution derives from the binomial law, although each component is a specified binomial distribution defined from the parameters of the multinomial distribution. But, it is still possible to interpret some of these components via a conditional Poisson distribution.

#### From binomial to multinomial distribution

The binomial distribution is a particular case of the multinomial distribution. We consider *S* a binomial distribution of parameters (*n*, *p*) counting the number of success of *n* independent events where the basic probability of success is *p*. Let *U* the random variable be defined by $n-S$ the number of failures. In the case where *S* represents the number of infections, *U* represents the number of uninfected places. The probability to get *k* infections is given by:

9 $$\begin{aligned} P(S = k) = {n \atopwithdelims ()k} p^k (1-p)^{n-k} = {n \atopwithdelims ()n-k} p^k (1-p)^{n-k} = P(U = n-k) \end{aligned}$$

As a result, the probability of having *k* success is the same that having $n-k$ failures. Then the Eq. (9) shows that *U* follows a binomial distribution with parameters $(n, 1-p)$. We can also say that the couple (*S*, *U*) follows a multinomial distribution of parameter $(p, 1-p, n)$, that we can denote $(S,U) \hookrightarrow {\mathcal {M}}(p, 1-p, n)$. In a more general way, the analogue of the binomial distribution is the multinomial distribution, where each trial results in exactly one of some fixed finite number *k* possible successes, with probabilities $p_1$, ..., $p_k$ (so that $p_i\ge 0$ for i = 1, ..., k and $\sum \nolimits _{i=1}^k p_i = 1$), and there are *n* independent trials. Then if the random variables $X_i$ indicate the number of times outcome number *i* is observed over the *n* trials, the vector $X = (X_1, \ldots , X_k)$ follows a multinomial distribution with parameters *n* and *p*, where $p = (p_1, \ldots , p_k)$, that we can also write ${\mathcal {M}}\left( p_1,\ldots ,p_n, N = k\right)$^55^.

#### From Poisson to multinomial distribution

We consider here a total number of successes (meaning in our example a number of spores that fall on a place and infect it) *X* being the sum of the infections due to $\omega$ different strains $X_i$ ($1\le i \le \omega$). If we consider that each $X_i$ follows a Poisson law of parameter $\lambda _i$ and that they are all independent, then *X* follows a Poisson law of parameter $\displaystyle \lambda = \sum \nolimits _{i=1}^{\omega } \lambda _i$. The distribution of each $X_i$ conditionally to $X = n$ is a binomial distribution ${\mathcal {B}}(n,\frac{\lambda _i}{\lambda })$. We can prove it for all variable $X_j$, with $j \in \{1,\ldots ,\omega \}$:

$$\begin{aligned} P\left( X_j = k \left| \right. \sum _{i=1}^{\omega } X_i = n \right) &= \frac{P \left( X_j = k,\displaystyle \sum \nolimits _{\begin{array}{c} i = 1 \\ i\ne j \end{array}}^{\omega } X_i = n-k \right) }{P \left( \displaystyle \sum \nolimits _{i = 1}^{\omega } X_i = n \right) } \\ &= \frac{P\left( X_j = k \right) P\left( \displaystyle \sum \nolimits _{\begin{array}{c} i = 1 \\ i \ne j \end{array}}^{\omega } X_i = n-k \right) }{P \left( \displaystyle \sum \nolimits _{i = 1}^{\omega } X_i = n \right) } \end{aligned}$$

that we obtain using the Bayes formula for conditioning and the use of independence between the $X_i$’s. Then we replace the probabilities by their Poisson values:

$$\begin{aligned} P\left( X_j = k \left| \right. \sum _{i = 1}^{\omega } X_i = n \right) &= \frac{e^{-\lambda _j}{\lambda _j}^k}{k!} \frac{e^{- \displaystyle \sum \nolimits _{\begin{array}{c} i = 1 \\ i\ne j \end{array}}^{\omega } \lambda _i}{\left( \displaystyle \sum \nolimits _{\begin{array}{c} i = 1 \\ i\ne j \end{array}}^{\omega } \lambda _i \right) }^{n-k}}{(n-k)!} \frac{n!}{e^{-\displaystyle \sum \nolimits _{i = 1}^{\omega } \lambda _i}{\left( \displaystyle \sum \nolimits _{i = 1}^n \lambda _i \right) }^{\omega }} \\ &= {n \atopwithdelims ()k} \frac{{\lambda _j}^k {\left( \displaystyle \sum \nolimits _{\begin{array}{c} i = 1 \\ i\ne j \end{array}}^{\omega } \lambda _i \right) }^{n-k}}{{\left( \displaystyle \sum \nolimits _{i = 1}^{\omega } \lambda _i \right) }^n} = {n \atopwithdelims ()k}{\left( \frac{\lambda _j}{\displaystyle \sum \nolimits _{i = 1}^{\omega } \lambda _i}\right) }^k {\left( \frac{\displaystyle \sum \nolimits _{\begin{array}{c} i = 1 \\ i\ne j \end{array}}^{\omega } \lambda _i}{\displaystyle \sum \nolimits _{i = 1}^{\omega } \lambda _i}\right) }^{n-k} \end{aligned}$$

Generalizing this result to the random vector of the $\displaystyle (X_i)_{1 \le i\le \omega }$ for $\omega$ strains, the distribution of this vector conditionally to the total number *X* is a multinomial distribution ${\mathcal {M}}\left( \frac{\lambda _1}{\lambda },\ldots ,\frac{\lambda _n}{\lambda }, X = n\right)$^55^.

### Properties of the model

Let $X_1$,..., $X_{2^{\nu }}$ independent random variables such that $X_j \hookrightarrow {\mathcal {P}}(\lambda _{{ infected},j}(t))$ for all $j \in \{1,\ldots ,2^{\nu }\}$, we have the following results:

A. When $$m \rightarrow \infty$$, $$\displaystyle N_{ infected}(t) \hookrightarrow {\mathcal {P}}(\sum \nolimits _{j=1}^{2^{\nu }} \lambda _{{ infected},j}(t))$$,

B. For all $$j \in \{1,\ldots ,2^{\nu }\}$$, $$\displaystyle N_{ infected, j}(t) \xrightarrow {\text {distribution}} X_j \left| \right. \sum \nolimits _{i = 1}^{2^{\nu }} X_i = n$$,

C. With A and B when $m \rightarrow \infty$, it is equivalent to either simulate $N_{ infected}(t)$ then the conditional multinomial vector $\displaystyle \left( N_{{ infected},1}(t), \ldots , N_{{ infected},2^{\nu }}(t)\right)$ conditionally to the realisation $n_{ infected}(t)$, or to simulate directly the previously defined variables $X_1$,..., $X_{2^{\nu }}$.

The number of infected sites due to strain *j*, without any loss of generalities, follows the binomial distribution $\displaystyle {\mathcal {B}}\left( n_{ infected}(t),\frac{\lambda _{{ infected},j}(t)}{\lambda _{{ infected}}(t)}\right)$. It is important to notice that it is the same law as a Poisson variable with parameter $\lambda _{{ infected},j}(t)$ conditionally to the realisation $n_{ infected}(t)$ of a Poisson variable with parameter $\lambda _{{ infected}}(t)$. Referring to formula (10), we can see that when the number of sites available for infection goes towards infinity, meaning that $N_{{ infected}}(t)$ behaves like a Poisson law of parameter $\sum \nolimits _{i = 1}^{2^{\nu }} \lambda _{{ infected},i}(t)$, then the variables $\displaystyle \left( N_{{ infected},i}(t)\right) _{1 \le i \le 2^{\nu }}$ behave like independent Poisson law of respective rates $\displaystyle \left( \lambda _{{ infected},i}(t)\right) _{1 \le i \le 2^{\nu }}$.

### Proof of the properties of the model

A. With the help of the reminder, we just have to prove this result:

10 $$\begin{aligned} \lim _{m \rightarrow \infty } E\left( N_{ infected}(t)\right) = \sum _{i=1}^{2^{\nu }} \lambda _{{ infected},i}(t), \end{aligned}$$

which could be obtained with the mean value theorem^56^. It means that if the total number of places available for infections was unlimited, these infections could be regarded as being Poisson distributed, with infection pressure as defined previously.

We consider the notation of (5), and to simplify the formula we will note: $\rho _i = \frac{1}{m} . \beta _i$ and because the result (10) does not depend on time we reduce the notation such that (10) is equivalent to:

11 $$\begin{aligned} \lim _{m \rightarrow \infty } E\left( N_{ infected}\right) = \sum _{i=1}^{2^{\nu }} \lambda _{{ infected}, i}, \end{aligned}$$

and then we want to prove that:

12 $$\begin{aligned} \lim _{m \rightarrow \infty } m . \left( 1 - \prod _{i=1}^{2^{\nu }} \left( 1- \frac{\beta _i}{m}\right) ^{n_{{ released},i}}\right) = \sum _{i=1}^{2^{\nu }} \lambda _{{ infected}, i} \end{aligned}$$

Replacing *m* by $\frac{1}{x}$, with $x\ne 0$, the latest equation equals:

13 $$\begin{aligned} \lim _{x \rightarrow 0} \frac{1}{x}. \left( 1 - \prod _{i=1}^{2^{\nu }} (1- x\beta _i)^{n_{{ released},i}}\right) = \sum _{i=1}^{2^{\nu }} \lambda _{{ infected}, i} \end{aligned}$$

We define $\displaystyle f_{\beta , n_{released}}(x) = \prod _{i=1}^{2^{\nu }} f_{i,({\beta , n_{released}})}(x) = \prod _{i=1}^{2^{\nu }} (1 - x\beta _i)^{n_{{ released},i}}$. Taking into account the fact that

14 $$\begin{aligned} f_{\beta , n_{released}}'(x) = \left( \prod _{i=1}^{2^{\nu }} f_{i,({\beta , n_{released}})}(x)\right) ' = \sum _{i=1}^{2^{\nu }} \left[ f_{i,({\beta , n_{released}})}'(x) \prod _{\begin{array}{c} i=1 \\ j\ne i \end{array}}^{2^{\nu }}f_{j,({\beta , n_{released}})}(x)\right] , \end{aligned}$$

we apply the mean value theorem (^56^) to the derivable function $f_{\beta , n_{released}}$, we got the following result:

15 $$\begin{aligned} \lim _{x \rightarrow 0} \frac{\left( 1 - f_{\beta , n_{released}}(x)\right) }{x} &= - \lim _{x \rightarrow 0} \frac{\left( f_{\beta , n_{released}}(0) - f_{\beta , n_{released}}(x)\right) }{0 - x} \nonumber \\ &= -\left( f_{\beta , n_{released}}'(0)\right) = \sum _{i=1}^{2^{\nu }} \beta _i n_{released, i} \end{aligned}$$

that finishes the proof of point A.

B. The result is immediate knowing the upper reminder concerning the Poisson–Multinomial laws relationship. We just have to take the value of $\omega = 2^{\nu }$.

C. When *m* is close to infinity, $N_{ infected}(t)$ follows a Poisson distribution whose parameter (expectation) is a sum of parameters. A property of Poisson distribution is that the law of a sum equals in distribution the sum of independent Poisson variables with the respective terms. So that we can rewrite B: For all $j \in \{1,\ldots ,2^{\nu }\}$, $\displaystyle N_{ infected, j}(t) \xrightarrow {\text {distribution}} X_j \left| \right. N_{ infected}(t) = n$.

## Results

Under the baseline parameters (Case 1), crop cultivar rotations greatly reduced the total amount of pathogen, compared to no rotation (S1, Fig. 3). With no rotation (S1), the grown cultivar selected the strain with the ‘matching’ gene of virulence, and this strain increased over the years until the whole field was infected. When rotation was made every year (S2), disease incidence was never significant, even though each virulence gene was quickly selected in turn (Fig. 3). At the end of the simulation, the four genes of virulence were at very high frequencies, indicating that a super-virulent strain with all four virulence genes had been selected for under this strategy. When rotation was made every 5 years (S3), disease incidence reached a peak just before the cultivar was changed, with the successful pathogen strain being the one that has the virulence gene required to ‘break’ the resistant gene (Fig. 2) of the deployed cultivar (S3, Fig. 3). Each virulence gene was selected in turn, with the deployment of the corresponding cultivar, and again a super-virulent strain with all four virulence genes had been selected for after 25 years, although the disease was hardly detectable. Selection of super-virulence was faster with 1-year (S2) than 5-year rotations (S3), but 1-year rotations (S2) maintained lower infection levels (Fig. 3). When two genes of virulence were stacked together (S4), the disease was eradicated after 5 years (S4; Fig. 3).

We then considered how model predictions for the four rotation scenarios would be affected by varying key model parameter values (Table 1), including the initial virulence allele frequency (*Init*.*freq*), the fitness modifier associated with each virulence allele ($\delta$), and the modifier of increase rate for non-virulent strains ($\epsilon$). We conducted a wide range of simulations with different combinations of parameter values, and then selected a few examples that were particularly informative for presentation here.

Increasing the initial virulence allele frequency ($\delta$) from $5\%$ to $50\%$ (Case 2) resulted in a few important differences (Fig. 4 cf Fig. 3). Without rotation (S1), infection by the non-corresponding pathogen genotypes were observable at first, until the non-corresponding alleles slowly faded out of the population (S1-left panel, Fig. 4). Moreover, the early peaks of infection in the 5-year rotation (S3) were higher. For all rotation strategies, now including the stacked gene strategy (S4), a super-virulence pathogen strain was selected, and infection levels by the super-virulent strain were now observable and highly significant after the first few years (Fig. 4). Selection of super-virulence was again faster with 1-year (S2) than 5-year rotations (S3), and faster still for the stacked gene strategy (S4). The 1-year rotations (S2) and the stacked gene strategy (S4) maintained lower infection levels than 5-year rotations (S3) at first, but reached higher levels after 25 years (Fig. 4).

Next, increasing the fitness penalty (decreasing $\delta$ from 0.9 to 0.7) generally decreased infection levels (Fig. 5 cf Fig. 4). In this scenario (Case 3), all rotation strategies were effective in eliminating the disease, with the eradication being fastest for the stacked gene strategy (S4), almost as fast for the 1-year rotations (S2), and slowest for the 5-year rotation (S3) (Fig. 5). The elimination of the disease occurred despite the fact that virulence genes had reached very high frequencies just prior to elimination occurring. The only observable infection, with exception of the no rotation strategy (S1), occurred in the 5-year rotation strategy (S3) (Fig. 5).

Subsequently, increasing $\epsilon$ from 0.05 to 0.5 (Case 4) resulted in only a small change to the predicted infection rates, but it did make a difference to virulence allele frequencies as expected (Fig. 6 cf Fig. 5). In all the rotation strategies, the virulence allele frequencies were pushed towards zero over time. While it may not be clearly evident in the graphs, in Fig. 6 the disease was completely eradicated in all the rotation strategies within 25 years, while in Fig. 5, the disease was not eradicated within 25 years in any of the rotation strategies. Instead, the virulence alleles were eradicated from the population, leaving only the non-virulent pathogen strain (i.e. 0000; Fig. 2) at relatively low levels. When all the parameter values were returned to baseline values ($\delta$ = 0.9, *Init*.*freq* = 0.05 and *Init*.*path* = $10\%$ of *m*), but $\epsilon$ was 0.5 instead of 0.05 (Case 5, Fig. 7), then results were similar to the baseline scenario (Fig. 3), except that selection of virulence was much slower and infection levels were somewhat higher.

## Discussion

### Overview of the model

We have developed a dynamic spatially implicit model to simulate the evolution of different pathogen strains within a field in response to different rotation strategies of resistant crop cultivars. The model represents the different phases of a polycyclic disease, including phases when the pathogen persists in crop residues (gene recombination during sexual reproduction and subsequent release of spores) or on living plants (asexual reproduction and secondary infections)^57^. Moreover, the model represents selection and competition between pathogen strains with a cultivar-pathogen interaction matrix while allowing for a fitness cost that sequentially penalises virulent strains with an increasing number of virulence genes^6,58,59^. The model includes intra-seasonal dynamics but ignores inter-seasonal variation for the sake of simplicity and generality, but it could also be extended to take into account seasonal differences, for example, climate variables and time of planting and harvesting. Lastly, the model achieves its aim of being capable of representing Blackleg disease, but it also offers the capability of being applied more generally to other diseases.

We believe the model is highly parsimonious in being one of the simplest possible models representing key processes of pathogen population and its evolutionary dynamics while accounting for sexual recombination and the fact that potential infection sites are limited^17,31,43,45,47,48^. The model achieves our aim of accounting for the important processes involved in the evolution of pathogen virulence over a number of seasons, without including additional parameters likely to increase model complexity and parametrisation sensitivity. Furthermore, the model is stochastic but consistent, providing very similar outcomes across different simulation runs with the same parametrisation. Only in a few ‘borderline’ cases is the stochasticity apparent; for example, in the baseline parameters case (Table 1) the stacked rotation strategy generally resulted in disease extinction (as illustrated in Fig. 3), but it occasionally resulted in selection of super-virulence and high infection levels instead. Lastly, the model is spatially implicit—it does not explicitly account for spatial structure in pathogen populations or cultivar deployment—but uses the surrogate *m* (total number of potential lesions, Table 1). We acknowledge that spatiotemporal dynamics are key when modelling disease epidemiology and evolutionary dynamics^6,10,31^ and we hope to implement such processes in future versions of the model.

### Overview of the simulation results

Our simulation results indicated that the population and evolutionary dynamics of the different pathogen strains are driven by a range of factors, including crop cultivar rotation strategies, fitness penalties and the degree that growth rates are reduced in the absence of virulence genes. Virulence evolution and infection levels over time are also highly dependent upon initial conditions such as the total amount of pathogen inoculum and the frequency of the different virulence alleles^6,60^.

In baseline simulations, when there was no rotation (S1) and the same cultivar was deployed every year, the pathogen strain with the exact virulence alleles to overcome the resistance alleles of the crop cultivar (see Fig. 2) was quickly selected for (Fig. 3). The more resistance genes the crop cultivar has, the greater the number of corresponding virulence genes that will be selected for. The speed of selection of the corresponding virulence genes depends on the degree to which avirulent strains (i.e pathogen strains without virulence genes) can grow and reproduce on the deployed cultivar, here determined by the $\epsilon$ parameter. For example, if $\epsilon$ was set to zero, selection occurred within a single season. The non-corresponding virulence alleles disappeared from the population slowly over time due to their fitness penalties. This meant that the only process occurring was then the bounded exponential growth of the corresponding pathogen strain. This growth period was extended when the cultivar deployed had more resistance alleles, due to the higher fitness penalty. Moreover, the initial virulent pathogen population was smaller due to the need for more virulence alleles to occur together in a single strain to confer effective virulence. The time taken for pathogen populations to build up in these no rotation baseline simulations (S1) also depended strongly on the initial population size and the initial virulence allele frequency.

The results clearly indicate the importance and possible effectiveness of rotating cultivars with different resistance genes. Regardless of the initial pathogen population level or the rotational strategy employed, rotation always kept pathogen populations lower for longer than deploying a single resistance gene cultivar every year. However, the efficacy of rotation depended strongly on the initial frequency of the virulence alleles. With the baseline parameter values (Case 1), pathogen populations were kept at negligible levels for at least 25 years in all tested rotation strategies (Fig. 3), despite the fact that a super-virulent strain with all virulence alleles was selected for well within 25 years for two of the strategies (S2 and S3; Fig. 3). While all three rotation strategies (S2-S4) worked well with lower initial virulence allele frequency (Fig. 3), the best strategy appeared to be the stacked gene strategy (S4), because it avoided selection of the super-virulent strain. On the other hand, for the scenario where virulence frequency as 0.5 instead 0.05 (Case 2, Fig. 4), the best strategy appeared to be the 5-year rotation strategy (S3), as it kept pathogen populations under control and also avoided selection of the super-virulent strain for longer (Fig. 4). Model analysis also highlighted the importance of fitness penalties and the degree to which growth rates are reduced in the absence of virulence genes that overcome the crop cultivars’ resistance genes (i.e. non-virulent pathogen strains) (e.g. Figs. 5, 6). Increasing the fitness penalty (i.e. reducing $\delta$ here) led to the disease being completely eradicated in all rotation strategies, even when the initial virulence frequency was high (Case 3 and 4, Figs. 5, 6). When we allowed the non-virulent pathogen strains to grow at a relatively higher rate on resistant crop stubble (Fig. 6), infection was maintained at low levels with a high fitness penalty, because the virulence genes were pushed to extinction, even though the disease was not completely eradicated.

In combination, these results highlight the difficulty of making general statements about the likely efficacy and/or relative advantage of different resistant cultivar rotation strategies. From a practical perspective, it has been demonstrated that growing cultivars with the same Blackleg resistance genes leads to changes in the pathogen’s virulence^6^. Such changes enable the pathogen to overcome cultivar Blackleg resistance. However, it is recommended to Australian canola producers that they can reduce the probability of resistance breakdown and reduce disease severity by simply rotating between cultivars with different resistance genes^61^. The lack of complete understanding of fitness penalties, current virulence allele frequencies, and to a lesser extent, the ability of non-virulent pathogen genotypes to grow and reproduce on resistant crop cultivars also add to difficulty to assert the efficacy of rotation strategies^6,17,31,58,59,62^. Our results showed that varying fitness penalty or the initial virulence allele frequency could totally change the efficacy of a given rotation strategy. For example, rotating cultivars with stacked resistance genes (S4) could eradicate the disease if initial virulence allele frequencies were relatively low (S4, Fig. 3), but quickly select for super-virulence if initial virulence allele frequencies were relatively high (S4, Fig. 6). In addition, varying the ability of non-virulent pathogen strains to grow and reproduce on resistant crop cultivars (i.e. $\epsilon$ parameter) could change whether the disease was eradicated (Fig. 6) or the rate at which virulence evolved (Fig. 7). In particular, our results highlight the importance of using crop cultivar rotation strategies consistently while pathogen virulence gene frequencies are still low, rather than introducing them once pathogen gene frequencies are high.

### Model assumptions and future improvements

Like all models, the current model is based on simplifying assumptions, some of which might be usefully broadened in future work. Multiple species could be introduced to allow investigation of crop species as well as resistant cultivars of a single crop. This could be done in either a spatial or non-spatially explicit way. A spatially explicit version of the model would allow us to investigate the effects of different strategies of crop and cultivar placement across a landscape in addition to rotation strategies through time^31,44,45^. The value added by and the need for such a spatially-explicit model would depend on the pathosystem type and the dispersal strategy (e.g. wind-borne, rain-splashed and/or soil-borne) and on the typical dimension of the fields and/or assemblage of fields in the situation being investigated^4,18,43,63^.

This study considered only four resistance genes, each with a major effect; in reality there may be more major resistance genes available^64,65^. These could be relatively easily added, but we would expect similar outcomes to the results observed here. Minor quantitative resistance traits could also be added, and the model could be extended to represent this kind of resistance as well. Furthermore, the current model does not represent ongoing mutation; this means that extinction of alleles can occur. In reality, extinction may be unlikely, due to ongoing mutation in combination with huge spore numbers leading to constant reintroduction of virulent alleles. We do not believe this is a significant caveat for this study, since adding mutation would just translate into frequencies being driven to very low levels rather than completely eradicated. Nonetheless, mutation could be added to future versions of the model, so that its effect can be explicitly studied and situations where mutation is crucial are properly accounted for in the future. Unlike many models of resistance evolution to xenobiotics, by representing a finite population with stochastic processes we have accounted for genetic drift^66,67^. Lastly, the way population dynamics are represented in the current model assumes that the size of the final pathogen population in a season depends on its size at the start of the season, which consequently depends on their size at the end of the previous season (Fig. 1). In some cases, when conditions between cropping seasons are particularly unfavourable for the pathogen, pathogen load seems to depend almost entirely on weather conditions during a given season rather than previous population size^68^. Thus, it may be useful to adapt the model to represent this situation in future as well.

### Implications of the simulation outcomes

Our results indicate that rotating cultivars with a number of single (S2 and S3) or stacked resistance genes (S4) may significantly delay the build-up of pathogen populations to damaging levels. The potential downside of rotation strategies is the selection of super-virulent pathogen strains. Even though we found cases where virulence alleles were completely eliminated (e.g. S4, Figs. 3, 7 and S2–S4, Figs. 5, 6), in reality, the reintroduction of virulence alleles through mutation and spatial dispersal from adjacent fields means that elimination is unlikely, and eventual emergence of a super-virulent strain is highly likely. Therefore, consideration should always be given to combining resistant cultivar rotations with additional crop management practices such as: i) one or more applications of pesticides; ii) adjusting the timing of sowing; iii) by spatial arrangement of crops; iv) by making tactical planting decisions based on weather conditions; and v) by strategies of rotation with alternative/non-host crops^10,31,61^. Nonetheless, the amount of time until the super-virulent strain causes populations to rise to damaging levels may be great enough, such that rotation of cultivars of the same crop is a viable strategy by itself; depending on the economics of the crop in question, and the availability of alternative/non-host crops.

We expect that current and future versions of the model will prove useful for predicting rotation strategies’ capacity to negate or at least halt resistance circumvention by pathogens in crop cultivars, and therefore minimise yield losses and maximise crop profitability. The model is general enough that it can be applied to identifying general strategies across a wide range of pathogens-crops combinations as well as management strategies without major modifications. We hope that this will help prevent or at least delay the collapses of major gene host resistances in cropping systens.
